# The Interconnected Mechanisms of Oxidative Stress and Neuroinflammation in Epilepsy

**DOI:** 10.3390/antiox11010157

**Published:** 2022-01-14

**Authors:** Anna L. M. Parsons, Eboni M. V. Bucknor, Enrico Castroflorio, Tânia R. Soares, Peter L. Oliver, Daniel Rial

**Affiliations:** 1 MRC Harwell Institute, Harwell Campus, Oxfordshire OX11 0RD, UK; a.parsons@har.mrc.ac.uk (A.L.M.P.); e.bucknor@har.mrc.ac.uk (E.M.V.B.); enrico.castroflorio@icfo.eu (E.C.); t.soares@har.mrc.ac.uk (T.R.S.); p.oliver@har.mrc.ac.uk (P.L.O.); 2 The Institute of Photonic Sciences, Parc Mediterrani de la Tecnologia, Av. Carl Friedrich Gauss, 3, 08860 Barcelona, Spain

**Keywords:** oxidative stress, epilepsy, seizure, synapse, neuroinflammation, neuron, astrocyte, neurotransmission

## Abstract

One of the most important characteristics of the brain compared to other organs is its elevated metabolic demand. Consequently, neurons consume high quantities of oxygen, generating significant amounts of reactive oxygen species (ROS) as a by-product. These potentially toxic molecules cause oxidative stress (OS) and are associated with many disorders of the nervous system, where pathological processes such as aberrant protein oxidation can ultimately lead to cellular dysfunction and death. Epilepsy, characterized by a long-term predisposition to epileptic seizures, is one of the most common of the neurological disorders associated with OS. Evidence shows that increased neuronal excitability—the hallmark of epilepsy—is accompanied by neuroinflammation and an excessive production of ROS; together, these factors are likely key features of seizure initiation and propagation. This review discusses the role of OS in epilepsy, its connection to neuroinflammation and the impact on synaptic function. Considering that the pharmacological treatment options for epilepsy are limited by the heterogeneity of these disorders, we also introduce the latest advances in anti-epileptic drugs (AEDs) and how they interact with OS. We conclude that OS is intertwined with numerous physiological and molecular mechanisms in epilepsy, although a causal relationship is yet to be established.

## 1. Introduction

Epilepsy is a group of heterogeneous diseases affecting 50 million individuals worldwide across all ages and ethnicities [1,2]. According to the International League Against Epilepsy (ILAE) and the International Bureau for Epilepsy (IBE), epilepsy is a disease of the brain characterized by an enduring predisposition to generate epileptic seizures [3], combined with the neurophysiological hallmark of hyperexcitability. Clinically, epilepsy is classified initially as generalised or partial, with further sub-classifications according to the seizure type and duration, overall severity and physical consequences to the patient [4]. To add to this complexity, differences in the underlying cause, neurophysiological and neuroimaging characteristics and comorbidities are also major players in disease prognosis and management [5]. Recent advances in genomic sequencing have offered new perspectives on the importance of genetic factors in epilepsy, now implicated in up to 40% of cases; however, a significant proportion (30%) are still classified as ‘of unknown cause’ (reviewed in Reference [6]). The remaining 30% are described as ‘acquired causes’ where primary events such as a stroke, brain tumours, head injuries (e.g., traumatic brain injury, encephalitis and degenerative disorders) precede epilepsy itself [7]. As such, the relatively limited nature of our knowledge regarding the etiology of epilepsy indicates that much progress is still needed. Firstly, around the identification and mechanistic understanding of new genetic causes, where multiple mutations in the same gene may provide novel genotype:phenotype correlations [8]. Secondly, regarding the underlying dynamics of neurotransmission and its mechanisms that are pivotal for disease management; indeed, many of the known genetic causes already cluster around fundamental aspects of neuronal cell communication [9].

It is widely accepted that seizures alter many chemical and biophysical processes in the central nervous system (CNS) and there are several reports indicating that the formation of reactive oxygen species (ROS) and the resulting cellular oxidative stress (OS) may play a role in seizure activity [10,11,12]. Significantly, the brain represents the largest source of energy consumption in the human body, accounting for over 20% of total oxygen metabolism [13]. Of this, it is estimated that neurons consume 75–80% of energy produced in the brain [14]; consequently, the brain is a prime target for OS.

To add another layer of complexity, epilepsy and OS are also linked to neuroinflammation [15]. For example, the involvement of pro-inflammatory cytokines such as interleukin 1-beta (IL-1β), interleukin-6 (IL-6) and tumour necrosis factor alpha (TNF-α) has been suggested to induce seizures [16] and high levels of these cytokines have been found in the cerebral spinal fluid (CSF) and blood serum of patients with epilepsy [17]. Furthermore, as reviewed elsewhere, the complete ‘neuroinflammatory machinery’—including glial and immune activation-mediated responses—seem to be associated strongly with epilepsy and OS-related mechanisms [18].

Despite our growth of knowledge in this area, the fundamental question of causality between these events remains unanswered. In this review, we aim to provide insight into the relationship between OS, neuroinflammation and epilepsy and the implications for synaptic function. We also discuss classical and new anti-epileptic drugs (AEDs) and their interference with OS, as well the influence of neuroinflammation and new strategies for the clinical management of epilepsy.

## 2. OS and Epilepsy

OS is generated from an imbalance between ROS production and clearance, ultimately causing potential damage to intracellular components via processes such as aberrant lipid peroxidation or protein oxidation [19]. Endogenous antioxidant systems, which are broadly classified as either enzymatic or non-enzymatic, have therefore evolved to maintain a safe OS balance [20].

Enzymatic antioxidant defence involves a neutralising chain of enzymes capable of reducing free radicals through electron scavenging to prevent the harmful build-up of ROS. The first of these enzymes, superoxide dismutase (SOD), contains either manganese, copper or zinc depending on cellular/extracellular location; these transition metals are capable of altering valence state to transfer electrons [21]. Following free radical formation, for example through mitochondrial respiration, SOD catalyses the superoxide anion, O_2_^−^, into hydrogen peroxide (H_2_O_2_), which is then scavenged and converted into water and oxygen by either catalase or glutathione peroxidase (GPx). Pertinent to epilepsy, mice lacking mitochondrial SOD (*Sod2−/−*) and heterozygous animals (*Sod2+/−*) show higher seizure susceptibility and concomitant degeneration, introducing the notion that O_2_^−^ are key players in the long-term brain changes after a seizure episode [22,23].

GPx is a selenoprotein, with a constituent selenium, which becomes oxidised and requires glutathione (GSH) to act as an electron acceptor, before being regenerated by GSH reductase in the presence of NADPH [24]. In patients, a prospective case-control study revealed that the serum levels of GPx are higher in epileptic patients in comparison to controls and that epilepsy mono- or polytherapeutics do not have any effect on this parameter, perhaps indicating why the current AED therapies do not efficiently control epilepsy-induced OS [25]. Increased cortical levels of GSH are seen following seizure-induced OS in the kainate model of epilepsy in rats, and GSH levels are negatively correlated with protein and lipid oxidation, supporting the role of GSH as a key antioxidant molecule [26].

Non-enzymatic antioxidants are electron-scavenging molecules, which are usually soluble and are able to traverse the body through the circulatory system. For example, albumin is a highly soluble protein with a flexible structure enabling extraordinary ligand binding ability. It contains a reduced cysteine residue, Cys34, which allows scavenging of hydroxyl radicals [27], and also binds metals such as copper, preventing ROS formation through the Fenton reaction. Albumin levels in *status epilepticus* (SE) recovering patients tend to be lower while its CSF/serum coefficient (a marker of blood-brain barrier disruption) were shown to be elevated [28,29]. These data suggest a compensatory mechanism where the albumin scavenging properties are utilised to minimize the effects of OS in the brain.

At the onset of OS, a transcription factor cascade begins that increases expression of antioxidant and protective genes. The antioxidant response element (ARE) is a *cis*-acting sequence found in the promoter regions of crucial detoxification enzymes [30]. Two genes in particular regulate ARE activation and expression, *Nrf1* and *Nrf2*, both from the Cap-N-Collar (CNC) gene family. Nrf1 activates expression of genes involved in GSH biosynthesis [31]; however, the gene appears to operate mostly at a basal level, whereas Nrf2 plays an inducible role in the transcription factor cascade. Double knockouts of *Nrf1* and *Nrf2* in mice show embryonic lethality and severe oxidative stress, suggesting overlapping roles between the two [32]. Through positively regulating ARE activity, Nrf2 induces expression of hundreds of antioxidant genes including GSH- and NADPH-dependent antioxidant enzymes, metal chelators such as ferritin and heme oxygenase-1 (HO-1), resulting in an efficient indicator and modulator of OS in neurodegeneration [33]. Nrf2 itself is dynamically regulated by Keap1, a cytoskeletally-bound adaptor protein that promotes ubiquitylation of the constitutively unstable Nrf2 during periods of oxidative balance. However, during periods of OS, Keap1 is inhibited and therefore unable to promote the degradation of Nrf2, enabling it to activate the ARE-mediated antioxidant cascade [34].

Wang and colleagues evaluated whether the Nrf2/ARE pathway is affected in a rat kindling model of epilepsy. Elevated levels of Nrf2/ARE and upregulation of two gene products (HO-1 and NAD(P)H quinone dehydrogenase 1 (NQO1)) were found, suggesting that after seizure induction, Nrf2/ARE responses are the first in line to prevent cell death through OS [35]. Later studies have gone on to expand on the importance of the Nrf2/ARE complex in epilepsy in a number of animal models such as pentetrazol [36], lithium-pilocarpine [37] and kainate [38].

Not all data are in line with the notion that OS is instigated prior to and during seizure activity. For example, reduced OS status has been reported in children with newly diagnosed epilepsy [11]. The authors reported no differences in GSH, SOD and malondialdehyde (MDA), a marker of lipid peroxidation levels in patients in comparison to healthy subjects. In summary, while it is undeniable that OS and epilepsy are connected, further information is needed to understand the underlying mechanisms and dynamics. It may be that the degree of pathological OS in epilepsy varies according to the cause and the severity of the episodes, explaining some of the apparent inconsistencies described in the literature.

## 3. Role of OS in Neuroinflammation and Epilepsy

Not only are ROS potentially damaging, but they also act as vital cell signalling molecules and signal transducers, playing a key regulatory role in neuronal development, excitability and synaptic plasticity [39]. However, under increasing levels of OS in the CNS, ROS can impel aberrant signal transduction and activation of redox-regulated transcription factors, such as mitogen-activated protein kinases (MAPKs), nuclear factor- kappa B (NF-κB) and activator protein 1 (AP-1), many of which play an essential role in inflammatory systems [40,41]. Furthermore, H_2_O_2_ is an efficient activator of cytoplasmic NF-κB [42], allowing for nuclear translocation and transcriptional activation of immunomodulatory target genes, such as pro-inflammatory cytokines TNF-α, IL-1β and IL-6 [41] which have been implicated in the promotion of neuronal excitability and ictogenic events [43].

In acquired epilepsies, such as post-traumatic epilepsy following brain injury, the resultant tissue and cellular damage unleashes a pathophysiological cascade promoting ROS production [44,45]. A subsequent accumulation of mitochondrial damage, lipid peroxidation and oxidative modifications, alongside the release of damage-associated molecular patterns (DAMPs), drives neuronal dysfunction and induces neuroinflammation at the site of injury [46]. In a bid to re-establish homeostasis, these elevated ‘danger signals’, often released by neurons at the developing epileptogenic site, instigate pro-inflammatory responses through detection by pattern recognition receptors (PRRs) such as Toll-like (TLRs) and Nod-like (NLRs) immune receptors [47]. High mobility group box protein 1 (HMGB1) is a common DAMP released through oxidative neuronal injury; HMGB1 cytoplasmic translocation initiates inflammasome activation and release into the extracellular space, a mechanism shown to be seizure provoking [48]. As a ROS defence system, resident CNS immune cells such as microglia express a host of PRRs (e.g., TLR4/RAGE receptors) that can detect ROS and/or pro-inflammatory cytokines for subsequent activation and infiltration at the site of injury [49,50]. The HMGB1/RAGE/TLR4 signalling pathway is a key player in a number of neurological disorders that do not result in seizures; however, it has been hypothesized that the presence of extracellular HMGB1 contributes to selective vulnerability of neuronal subpopulations (e.g., pyramidal and granule cell neurons) to hyperexcitability and synaptic dysfunction within the inflammatory environment, further augmenting seizure activity [48,51]. Active secretion of HMGB1 from localised activated microglia and astrocytes readily increase the pool of this key DAMP [18], thereby creating neurotoxic feedback loop driving downstream ictogenic effects such as modulating synaptic plasticity, increasing blood brain barrier permeability and enhancing mossy fibre spouting [52]. This pathophysiological cascade further increases pro-inflammatory cytokine production and OS, priming the epileptogenic environment for initial and successive seizure activity.

Evidence of OS playing a direct role in the induction of neuroinflammation has been demonstrated in a pilocarpine-treated rat model of SE. Following treatment with MnIIITDE-2-ImP5+, a catalytic antioxidant with the ability to scavenge ROS, significant reductions in TNF-α and IL-1β levels were reported in the hippocampus—in addition to IL-6 and KC/GRO in the piriform cortex—compared to pilocarpine-only treated rats [53], thus demonstrating directly that ROS can modulate pro-inflammatory cytokine production post-seizure. In a pentylenetetrazol (PTZ) kindling rat seizure model, suppression of the HMGB1/RAGE/TLR4 inflammatory cascade by administration of pentoxifylline resulted in reduced ROS levels, whilst also improving cognition, memory and hippocampal neuronal survival [54], suggesting that regulation of neuroinflammatory pathways has a positive downstream effect on OS. However, this concept cannot be expanded to all epilepsies; using a rat model of audiogenic seizures, de Deus and colleagues reported similar levels of IL-6, IL-10, TNF-α and IL-1β between controls and experimental animals, but high correlation between seizure severity and nitrate levels [55].

A range of biomarkers for neuroinflammation has been identified in a host of epilepsy disorders [43,56,57]. Plasma levels of pro-inflammatory cytokines, including TNF-α, IL-6, and IL-1β were significantly upregulated in temporal lobe epilepsy patients [58]. Additionally, Ethemoglu et al. conducted a comparative study of biomarkers present in patients with drug-resistant epilepsies. Elevated levels of IL-6 and an overall higher oxidative stress index was observed in these patients compared to healthy controls or patients with ‘well-controlled’ epilepsy [59], potentially indicating that IL-6 might be a good prognostics marker for drug-resistant epilepsy.

Together these findings highlight the fact that, regardless of initial insult, there is an intricate crosstalk between OS and neuroinflammatory response in the CNS associated with seizure activity, further highlighting their potential as prominent targets for therapeutics in epilepsy.

## 4. Excitatory/Inhibitory Imbalance: Relevance to OS and Epilepsy

During development, the neuronal network endures extensive changes in connectivity that can threaten to destabilise its activity. Under normal physiological circumstances, homeostatic mechanisms act upon neurons to prevent hyper- or hypo-activation [60,61]. From the synaptic perspective, epilepsy involves an array of aberrant physiological alterations, such as mechanisms related to synaptic vesicle (SV) release [62,63,64], ion channel physiology [65,66] and energetic metabolism [67,68], resulting in an imbalance between excitation and inhibition in localized regions or multiple brain areas. These changes lead to morphological alterations of synaptic boutons, including the number of active zones and the number of SVs in the reserve pool and readily releasable pool [69,70,71]. At the molecular level, these changes can also affect chromatin remodelling [72,73], protein synthesis [74] and other important cellular processes such as autophagy [75]. For example, Hoffmann and colleagues used a light-activated ROS generator linked to SV proteins and discovered that ROS production is able to facilitate the removal of key regulators of synaptic transmission, such as synaptophysin, and this response was sufficient to induce autophagy [76]. In addition, mechanisms that limit the action of neurotransmitters, such as the expression of high affinity uptake transporters, are fundamental to the control of synaptic homeostatic balance. Membrane-bound sodium-dependent transporters such as the excitatory amino acid transporters (EAATs) and the gamma-amino butyric acid (GABA) transporters are essential to synaptic balance [77] and its function significantly modified in epilepsy [78,79].

Due to the imbalance of excitatory/inhibitory (E/I) mechanisms in epilepsy acting as the major trigger for seizure activity, the involvement of the excitatory neurotransmitter glutamate and the inhibitory neurotransmitter GABA is fundamental [80]. A key finding that attests to a glutamate-induced hyperexcitable state in epilepsy is the increased extracellular glutamate levels in experimental models of epilepsy and in the brains of epileptic patients [81,82]. Importantly, excessive stimulation of glutamate receptors contributes to the generation of free radicals by increasing the intracellular levels of calcium (Ca^2+^), a cofactor for nitrous oxide (NO) formation. Subsequently, this results in an imbalance of mitochondrial activity, ultimately facilitating synaptic malfunction [75]. In tandem, the excessive stimulation of glutamate receptors induces metabolic consequences, culminating in a change in receptor profile and dynamics [83].

In fact, evidence indicates that the entire glutamate machinery participates in epilepsy; from synthesis [84], receptor activity and associated signalling [85], to uptake [86]. N-methyl-D-aspartate glutamate receptors (NMDAR) play vital roles in memory formation and synaptic plasticity but also mediate increased Ca^2+^ conductivity, leading to excitotoxicity [87]. The NMDAR are composed of different combinations of NR1, NR2A and NR2B subunits and it has been shown that NR1 and NR2B are overexpressed in the hippocampus from a lithium chloride-pilocarpine chronic rat epilepsy model [88]. The indication is that not only are the number of receptors available in the membrane modified in this model, but also that their composition is altered. This example reveals that the chronic effect of seizures is to induce fundamental changes to the synaptic system, promoting increased tonic activation of glutamatergic neurotransmission. This implication is two-fold: first, contributing to hyperexcitation, and second, providing additional substrates for ROS formation and the induction of OS as a positive feedback mechanism.

α-Amino-3-hydroxy-5-methyl-4-isoxazolepropionic acid glutamate receptors (AMPAR) are dynamic receptors acting at the postsynaptic membrane. Reports have shown that AMPAR contribute to neuronal hyperexcitability and epileptogenesis by driving circuitry re-wiring [89], representing a novel target for epilepsy treatment [90]. Of interest, Levite et al. reported that the AMPA GluR3B receptor binds peptide autoimmune antibodies, inducing ROS production and necrosis in both human neuronal cells and T cells [91], indicating that the aforementioned receptors may play a functional role that links neuroinflammation and epilepsy [90].

It is becoming apparent that astrocytes are critical mediators of epileptiogenesis, in particular due to their tight coupling via gap junctions that facilitates rapid signaling responses during hyperactivity [92,93]. For example, a key aspect of glutamate physiology associated with epilepsy and OS is the neuron-astrocyte interaction. Astrocytes are responsible for the uptake of glutamate from the synaptic cleft, and in temporal lobe epilepsy (TLE), astrocyte-mediated glutamate clearance via the astrocytic glutamate transporter (GLT-1) is defective, resulting in glutamate accumulation and excitotoxicity [94,95]. Other well-studied and important contributions of astrocytes to synaptic dysfunction in epilepsy are astrocyte-mediated impaired potassium (K^+^) buffering, aquaporin (AQP4) dysfunction and excessive astroglial ATP release and purine receptor activation (reviewed in [93]). The activation of astrocytic purinergic receptors induces Ca^2+^-mediated signalling that could promote astrocytic release of gliotransmitters like glutamate or ATP, which ultimately would contribute to neuronal excitation (reviewed in [96]). Additionally, there is evidence for cytokine (TNF-α)-driven, autocrine astrocyte purinergic signaling that helps stimulate excitatory synaptic activity in the hippocampus in a model of TLE [97]; crucially, blocking this particular cytokine-associated pathway prevents aberrant glutamatergic gliotransmission and prevents hyperactivity [97].

The contribution of inhibitory GABA to seizure activity in epilepsy is equally as important as excitatory glutamate. GABA acts through the activation of GABA_A_ (a ligand-gated ion channel) and GABA_B_ (a G-protein-coupled channel) receptors. GABA_A_ activation leads to a rapid increase in the flow of chloride post-synaptically and is the base of action of first choice antiepileptic drugs such as barbiturates and benzodiazepines [98]; while antagonists of GABA_A_, such as bicuculline and picrotoxin, are proconvulsivants [99]. In humans, GABA concentrations or GABA-receptor densities have been found to be reduced in epileptic patients [100,101], and reduced binding to benzodiazepines has been demonstrated in the mesial temporal lobe on positron emission tomographic scanning [102,103] meaning reduced inhibitory capacity and responsiveness to pharmacological activation.

In respect to OS and epilepsy, GABA’s activity in the synapse appears to be strongly controlled by the presence of ROS. Several studies indicate that the presence of H_2_O_2_ or NO reduces the efficiency of GABA_A_ receptors, resulting in increased excitability [104,105,106]. Additionally, H_2_O_2_ provokes changes in the membrane and inhibitory synaptic properties; Frantseva et al. observed a significant reduction of inhibitory postsynaptic potentials (IPSP) by administration of H_2_O_2_ in hippocampal, thalamic and cortical rodent brain slices, reinforcing the interference of ROS in the inhibitory tonus [107].

**Figure 1:**
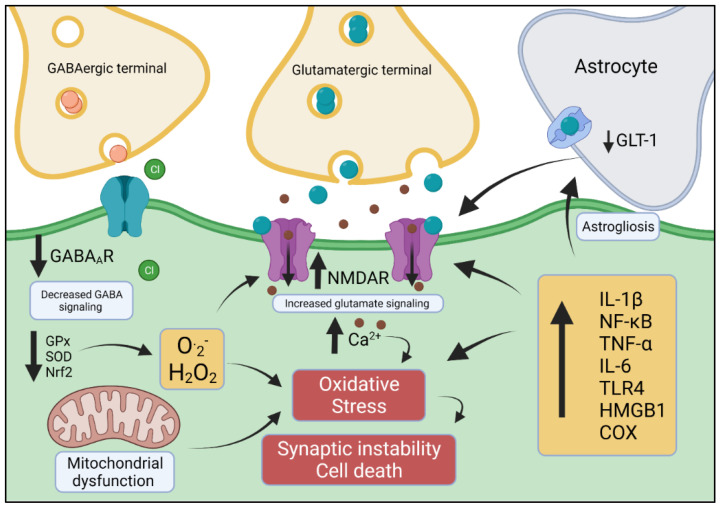
Interconnected mechanisms of epilepsy involving OS. Reduced GABA-mediated inhibitory responses paired with increased glutamatergic tonus (through NMDA currents and/or astrocyte clearance dysfunction) leads to increased intracellular calcium concentration, which is the main source of hyperexcitability as well as OS-associated feedback. In addition, a reduction in the expression of antioxidant defence proteins (for example, GPx, SOD and Nrf2) have been reported in epileptic patients and in animal models of epilepsy. The increased formation of ROS and consequent mitochondrial dysfunction contribute synergistically to OS resulting in synaptic malfunction and cell death. These events are all supplemented by neuroinflammation (e.g., indicated by increase cytokine expression) that potentiates OS and induces astrogliosis, in-turn impacting NMDA function and causing cell death.

Of note, the coordinated action of other neurotransmitters and neuromodulators (e.g., adenosine, dopamine and acetylcholine) is also essential to a finely tune the E/I balance (reviewed in [108]); however, here we have focused on the fundamental downstream responses directly influencing glutamate and GABA. Together, it is clear that the E/I equilibrium is altered in epilepsy and that OS plays an additional modulatory role, both increasing excitatory responses while decreasing inhibition (summarised in Figure 1).

## 5. Pharmacological Evidence of the Interaction between OS, Inflammation and Epilepsy

### 5.1. AEDs and OS

Recurrent seizures are known to induce neurodegeneration and contribute significantly to progressive cognitive decline, even in the presence of adequate treatment with AEDs. Importantly, around 30% of patients suffer from drug refractory epilepsy, indicating the need for new or additional therapeutic strategies [109]. The canonical mechanisms of AED action aim to decrease neuronal excitability by facilitating inhibitory responses or regulating channel conductance to decrease the bursting threshold of neurons [110]. Interestingly, some classical AEDs show surprising antioxidant or pro-oxidant properties [111], reinforcing the mechanistic overlap between epilepsy and OS. Indeed, protection against OS and the resultant neuronal injury may be crucial for the long-term success of current AEDs.

The known, but wide-ranging, effects of classical AED on oxidative stress markers are listed in Table 1. While some act on mitochondrial redox activity (Lamotrigine), others reduce lipid peroxidation (benzodiazepines, carbamazepine and barbiturates) independently of their ‘main’ mechanism of action. It is still to be defined whether this variety of antioxidant properties are particular characteristics of each drug, or if it reflects the different types of epilepsy or animal models studied that induce variable responses. It is also important to mention that some AEDs such as Valproic acid and Phenytoin increase lipid peroxidation or reduce the antioxidant capacity respectively; however, it is still unclear if these effects restrict their clinical efficacy.

### 5.2. Antioxidants with Anti-Epileptic Properties

Antioxidant strategies for treating neurological disorders such as epilepsy show some promise, either as monotherapy or in combination with existing AEDs. Despite this being a relatively new field, due to the epidemiological interest in the treatment of epilepsy, new molecules with improved pharmacological properties are continually being evaluated in the clinic. One very promising molecule with antioxidant effects and anti-epileptic activity is the phytocannabinoid cannabidiol. Combined with a multi-target anti-seizure mechanism, it not only promotes increased antioxidant defences but also reduces ROS production (Table 1). However, considering the previously mentioned complex balance of ROS and OS in brain function, the evaluation of this approach in other forms of epilepsy and pre-clinical models warrants further investigation. Moreover, the use of antioxidants therapeutically to enhance neuroprotection in epilepsy has had limited effectiveness for a number of reasons; many therapies only target one source of OS, and some antioxidants are location-specific. For example, although GSH acts as a crucial antioxidant molecule in the cortex, neuroprotection against OS in the hippocampus is dependent on SOD, therefore enhancing GSH levels would have little to no effect on epilepsies originating from the this brain region [26].

Although not an antioxidant molecule, one of the most effective antioxidant strategies to treat epilepsy is the use of the ketogenic diet (KD). KD has long been used to treat drug-resistant childhood epilepsy, yet only recently have its mechanisms of action, including antioxidant effects, begun to emerge. The diet contains high fat, low carbohydrate and controlled protein content, and studies report a reduction in seizure frequency up to 75% in epileptic children [140] and in mice lacking the voltage-gated K^+^ channel Kv1.1 [141]. KD increases physiological levels of long chain fatty acids, which are endogenous ligands of the transcription factor PPARγ2. It was recently discovered that the downstream effector of PPARγ2, the antioxidant enzyme catalase, is responsible for the KD’s efficacy in treating seizures, and furthermore reducing ROS burden [142]. In addition, the KD suppresses OS through the activation of OS-resistance genes, more specifically, the forkhead box O3a (*Foxo3a*) and metallothionein 2 (*Mt2*), which are upregulated by the ketone body β-hydroxybutyrate [143]. Consequently, the anti-seizure and antioxidant effects of the KD are inextricably linked.

### 5.3. Anti-Inflammatory Drugs with Anti-Epileptic Properties

As mentioned, the intricate crosstalk between OS and the neuroinflammatory response in the CNS is a well-established feature of epilepsy pathology, thus highlighting their potential as synergistic therapeutic targets. While some anti-inflammatory drugs associated with classical mechanisms—such as the inhibition of cyclooxygenases (aspirin)—are listed as drugs to treat epilepsy, their modest effects are over-shadowed by a new generation of anti-inflammatory/anti-epileptic therapeutic options (see Table 1).

Antibodies against key neuroinflammatory regulators such as TNF-α (adalimumab), IL-6 (tocilizumab) and IL-1 (canakinumab in combination with anakinra), reported promising results, reducing seizure frequency, severity and increasing the patients’ quality of life. One of the advantages of designed antibodies in comparison to classical drugs is the enhanced selectivity profile and specificity. Unlike classical drugs, antibodies bind to precise and exclusive epitopes, with the potential to avoid unwanted off-target side-effects. In summary, similarly to the application of antioxidants, preclinical and clinical testing of anti-inflammatory drugs against epilepsy is a new field, with factors such as long-term use still to be assessed.

## 6. Future Directions

Due to the detrimental effects of ROS exerted through the oxidation of essential molecules such as enzymes and cytoskeletal proteins [144,145], a therapy based on the elimination of excess ROS is potentially a tractable approach. However, displacement from the normal cellular redox state (termed ‘oxidative eustress’) to a reduced state is also detrimental [146]. As such, attempts to manipulate endogenous antioxidants for therapeutic benefit has proved to be challenging, in part due to the sensitivity of the ROS-associated homeostatic mechanisms, but also from issues around penetration of the blood-brain-barrier. One novel approach is to target key OS effectors using small molecules, such as the ARE/Nrf2 axis (see above), that form potentially long-lasting antioxidant signalling cascades. Critically, Nrf2-driven transcriptional activity is negatively regulated by Kelch-like ECH associated protein 1 (KEAP1), whereby KEAP1 binding prevents Nrf2 translocation to the nucleus [147]. Thus KEAP inhibitors such as Sulforaphane (see Table 1) are an attractive upstream target, although the lack of selectivity of this particular compound has limited further studies [148]. As an alternative, much more highly potent and specific Nrf2 activators have been studied, including the bardoxolone methyl analogue RTA 408, that has been shown to activate Nrf2 via KEAP1 inhibition [149]. In a recent promising in vivo study, RTA 408 administration after kainite-induced SE in the rat increases glutathione and ATP in the brain as well as preventing neuronal cell death. Furthermore, the same treatment results in an almost 10-fold reduction in spontaneous seizures at 4 months following SE [149]. In addition, in vitro molecular studies regarding the mechanism of action revealed that RTA 408 binds via a specific amino acid (C151) of KEAP1, thus preventing the interaction with Nrf2 [149]. Questions still remain regarding the longevity of such transcriptional modulators in patients, yet data from a recent clinical trial of the same compound in Friedrich’s Ataxia (FA), marketed as Omaveloxolone, are promising, showing significantly improved neurological function compared to placebo alongside no overt side-effects [150].

There will likely be future interest in combination therapies, where a general OS-related target will be modified in tandem with a known disease pathway or gene-specific therapeutic approach. Indeed, RTA 408 administration has been coupled with inhibition of the ROS producing enzyme NADPH oxidase (NOX) using 4-(2-aminoethyl)-benzenesulfonyl fluoride (AEBSF) in the same kainite-induced SE model as above. Significantly, the combined approach was synergistic, with 70% of animals rendered seizure free after 8 weeks of treatment [151]. The on-going clinical trials of Omaveloxolone in FA will hopefully facilitate future human studies, alongside further efficacy testing in vivo using well-established genetic models of epilepsy.

## 7. Conclusions

Despite significant advances in the genetics of epilepsy, the heterogeneity of disorders characterised by seizures continues to hamper the search for novel therapeutic strategies that will target multiple patient groups. One approach is to combine established AEDs with therapies aimed at antagonistic cellular features, such as OS and neuroinflammation. Here, we have described examples where OS is undoubtedly closely associated with seizure propagation, although in many cases ROS appear to be induced after localized hyperexcitability and are not a primary cause of disease. In particular, it is clear that the cytotoxicity that occurs downstream of ROS generation is not an absolute requirement for epileptogenesis; yet this phenomenon will certainly exacerbate aberrant network connectivity and subsequent neurodegeneration. Moreover, despite multiple examples of antioxidant-targeting treatments showing some promise in the mitigation of seizures, their apparent anti-convulsant properties are not yet proven to be driven by preventing OS in specific cellular populations. As such, there is much more to learn mechanistically regarding the ‘cause-and-effect’ and clinical efficacy of these approaches [152,153]. In addition, how such strategies, often tested in acute seizure-induction models, will be translated into the heterogeneous population of epilepsy patients remains to be shown.

On a more positive note, there are new examples that demonstrate targeting of broadly applicable OS-associated pathways—combined with improvements in CNS delivery—that show promise for the amelioration of seizures (see above). Indeed, a plausible aim for the field is to utilise such strategies as part of longer-term disease management to delay the often inevitable pathological and cognitive consequences of recurrent seizures [154]. The exact role of such complex comorbid conditions to altered AED response over time is unclear, although the longer-term, concomitant detrimental effects of OS, neuroinflammatory and synaptic dysfunction are common targets that could be further exploited therapeutically. In summary, there is still much to learn regarding the complex and interconnecting pathways that link OS, neuroinflammation and aberrant neurotransmission; further studies into these closely-related pathways provide promise for identifying novel therapeutics in the future.

## Figures and Tables

**Table 1:** Pharmacological effects of classical AEDs, antioxidants and anti-inflammatory compounds on epilepsy, OS and inflammatory markers.

Class	Drug	Mechanism of Action	Type of Seizure Targeted	Effects on OS and Inflammation Markers in Epilepsy (Pre- and Clinical Data)	References
Classical anti-epileptic	Valproic acid	Blocks voltage-gated ion channels	Focal and generalized	Increased lipid peroxidation	[111]
	Phenytoin	Blocks voltage-gated sodium channels	Tonic-clonic	Reduced antioxidant capacity and glutathione concentration; Increased lipid peroxidation	[112,113]
	Carbamazepine	Blocks sodium channels	Focal and generalized	Decreased lipid peroxidation; Increased NO release	[114]
	Barbitures	Potentiates GABA signalling	Generalized	Decreased lipid peroxidation; Reduced levels of antioxidant enzymes	[115]
	Benzodiazepines	Facilitates GABA binding to GABA_A_ receptors	Status epilepticus	Decreased lipid peroxidation	[116]
	Cenobamate	Blocks voltage-gated sodium channels. Allosteric agonist of GABA receptors	Uncontrolled focal	Activation of the PI3K/Akt-CREB-BDNF pathway	[117,118]
	Lamotrigine	Binds to the inactive sodium channel	Focal and generalized	Increased antioxidant defence; Reduced mitochondrial redox activity	[119]
Antioxidant	Cannabidiol	Inhibits GRP55; Desensitizes receptor potential vanilloid type-1; Inhibits adenosine uptake,	Drug-resistant	Decreased ROS production; Increased antioxidant defences	[120,121,122]
	Naringenin	Free radical scavenger	Pilocarpine-induced	Increased glutathione and antioxidant enzymes levels	[123]
	Coenzyme Q10	Increases the levels of TCA and antioxidant enzymes	Pilocarpine-induced	Increased SOD and GSH levels, reduced lipid peroxidation	[124]
	N-acetylcysteine	Reduces glutathione precursor	Pentylenetetrazole-induced	Attenuated the impairment in glutathione homeostasis	[125]
	Curcumin	Free radical scavenger and metal chelator	Pentylenetetrazole-induced	Increased superoxide dismutase levels Reduced the expression of inflammatory cytokines and chemokines Reduced GFAP and IBA-1 markers	[126]
	Vitamin E	Peroxyl radical scavenger	Refractory	Increased antioxidant capacity. Increased catalase and glutathione levels	[127]
	Sulforaphane	Activates NRF2/ARE pathway	Status epilepticus	Decreased malondialdehyde levels and increased glutathione levels	[128]
Anti-inflammatory	Anakinra	Antagonist of IL-1 receptor	Febrile infection- related epilepsy syndrome and Intractable epilepsy	Reduced IL-1 driven systemic autoinflammation	[129,130,131,132]
	Anakinra + Canakinumab	Antagonist of IL-1 receptor; Monoclonal antibody against the IL-1 receptor	Generalized		
	Tocilizumab	Anti-IL-6 monoclonal antibody	Status epilepticus, acute epilepsy	Reduced IL-6 levels	[133,134]
	Minocycline	Inhibitor of microglia activation	Drug-resistant	Supressed IL-1β release from microglia	[135]
	Adalimumab	Anti-TNF monoclonal antibody	Partial and focal motor seizures	Reduced TNF-α levels	[136]
	Aspirin	Cyclooxygenase inhibitor	Focal-onset	Not reported	[137,138]
	VX09-765-401	IL-1β inhibitor	Partial seizures	Not reported	[139]

## References

[1] World Health Organisation Epilepsy. www.who.int/news-room/fact-sheets/detail/epilepsy.

[2] Devinsky O., Vezzani A., O’Brien T.J., Jette N., Scheffer I.E., de Curtis M., Perucca P. (2018). Epilepsy. Nat. Rev. Dis. Primers.

[3] Fisher R.S., van Emde Boas W., Blume W., Elger C., Genton P., Lee P., Engel J. (2005). Epileptic seizures and epilepsy: Definitions proposed by the International League Against Epilepsy (ILAE) and the International Bureau for Epilepsy (IBE). Epilepsia.

[4] Stein M.A., Kanner A.M. (2009). Management of newly diagnosed epilepsy: A practical guide to monotherapy. Drugs.

[5] Pennell P.B. (2020). Unravelling the heterogeneity of epilepsy for optimal individualised treatment: Advances in 2019. Lancet Neurol..

[6] Perucca P., Bahlo M., Berkovic S.F. (2020). The Genetics of Epilepsy. Ann. Rev. Genom. Hum. Genet..

[7] Hauser W.A., Annegers J.F., Kurland L.T. (1993). Incidence of epilepsy and unprovoked seizures in Rochester, Minnesota: 1935–1984. Epilepsia.

[8] Carvill G.L., Matheny T., Hesselberth J., Demarest S. (2021). Haploinsufficiency, dominant negative, and gain-of-function mechanisms in epilepsy: Matching therapeutic approach to the pathophysiology. Neurotherapeutics.

[9] Fukata Y., Fukata M. (2017). Epilepsy and synaptic proteins. Curr. Opin. Neurobiol..

[10] Yang N., Guan Q.W., Chen F.H., Xia Q.X., Yin X.X., Zhou H.H., Mao X.Y. (2020). Antioxidants Targeting Mitochondrial Oxidative Stress: Promising Neuroprotectants for Epilepsy. Oxid. Med. Cell. Longev..

[11] Yiȿ U., Seςkin E., Kurul S.H., Kuralay F., Dirik E. (2009). Effects of epilepsy and valproic acid on oxidant status in children with idiopathic epilepsy. Epilepsy Res..

[12] Morimoto M., Satomura S., Hashimoto T., Ito E., Kyotani S. (2016). Oxidative stress measurement and prediction of epileptic seizures in children and adults with severe motor and intellectual disabilities. J. Clin. Med. Res..

[13] Magistretti J.P., Allaman I. (2015). A cellular perspective on brain energy metabolism and functional imaging. Neuron.

[14] Hyder F., Fulbright R.K., Shulman R.G., Rothman D.L. (2013). Glutamatergic function in the resting awake human brain is supported by uniformly high oxidative energy. J. Cereb. Blood Flow Metab..

[15] Alyu F., Dikmen M. (2017). Inflammatory aspects of epileptogenesis: Contribution of molecular inflammatory mechanisms. Acta Neuropsychiatr..

[16] Mlodzikowska-Albrecht J., Steinborn B., Zarowski M. (2007). Cytokines, epilepsy and epileptic drugs—Is there a mutual influence?. Pharmacol. Rep..

[17] Vezzani A., Granata T. (2005). Brain inflammation in epilepsy: Experimental and clinical evidence. Epilepsia.

[18] Vezzani A., French J., Bartfai T., Baram T.Z. (2011). The role of inflammation in epilepsy. Nat. Rev. Neurol..

[19] Cobb C.A., Cole M.P. (2015). Oxidative and nitrative stress in neurodegeneration. Neurobiol. Dis..

[20] Espinós C., Galindo M.I., García-Gimeno M.A., Ibáñez-Cabellos J.S., Martínez-Rubio D., Millán J.M., Rodrigo R., Sanz P., Seco-Cervera M., Sevilla T. (2020). Oxidative stress, a crossroad between rare diseases and neurodegeneration. Antioxidants.

[21] Marklund S.L. (1982). Human copper-containing superoxide dismutase of high molecular weight. Proc. Natl. Acad. Sci. USA.

[22] Liang L.P., Waldbaum S., Rowley S., Huang T.T., Day B.J., Patel M. (2012). Mitochondrial oxidative stress and epilepsy in SOD2 deficient mice: Attenuation by a lipophilic metalloporphyrin. Neurobiol. Dis..

[23] Liang L.P., Patel M. (2004). Mitochondrial oxidative stress and increased seizure susceptibility in Sod2 (−/+) mice. Free Radic. Biol. Med..

[24] Rotruck J.T., Pope A.L., Ganther H.E., Swanson A.B., Hafeman D.G., Hoekstra W.G. (1973). Selenium: Biochemical role as a component of glutathione peroxidase. Science.

[25] Keskin Guler S., Aytac B., Durak Z.E., Gokce Cokal B., Gunes N., Durak I., Yoldas T. (2016). Antioxidative-oxidative balance in epilepsy patients on antiepileptic therapy: A prospective case-control study. Neurol. Sci..

[26] Gluck M.R., Jayatilleke E., Shaw S., Rowan A.J., Haroutunian V. (2000). CNS oxidative stress associated with the kainic acid rodent model of experimental epilepsy. Epilepsy Res..

[27] Gutteridge J.M.C. (1986). Antioxidant properties of the proteins caeruloplasmin, albumin and transferrin a study of their activity in serum and synovial fluid from patients with rheumatoid arthritis. Biochim. Biophys. Acta.

[28] Correale J., Rabinowicz A.L., Heck C.N., Smith T.D., Loskota W.J., DeGiorgio C.M. (1998). Status epilepticus increases CSF levels of neuron-specific enolase and alters the blood-brain barrier. Neurology.

[29] Tumani H., Jobs C., Brettschneider J., Hoppner A.C., Kerling F., Fauser S. (2015). Effect of epileptic seizures on the cerebrospinal fluid–A systematic retrospective analysis. Epilepsy Res..

[30] Favreau L.V., Pickett C.B. (1991). Transcriptional regulation of the rat NAD(P)H: Quinone reductase gene. J. Biol. Chem..

[31] Kwong M., Kan Y.W., Chan J.Y. (1999). The CNC basic leucine zipper factor, Nrf1, is essential for cell survival in response to oxidative stress-inducing agents. J. Biol. Chem..

[32] Leung L., Kwong M., Hou S., Lee C., Chan J.Y. (2003). Deficiency of the Nrf1 and Nrf2 transcription factors results in early embryonic lethality and severe oxidative stress. J. Biol. Chem..

[33] Johnson J.A., Johnson D.A., Kraft A.D., Calkins M.J., Jakel R.J., Vargas M.R., Chen P.C. (2008). The Nrf2-ARE pathway: An indicator and modulator of oxidative stress in neurodegeneration. Ann. N. Y. Acad. Sci..

[34] Nguyen T., Sherratt P.J., Nioi P., Yang C.S., Pickett C.B. (2005). Nrf2 controls constitutive and inducible expression of ARE-driven genes through a dynamic pathway involving nucleocytoplasmic shuttling by Keap1. J. Biol. Chem..

[35] Wang W., Wang W.P., Zhang G.L., Wu Y.F., Xie T., Kan M.C., Fang H.B., Wang H.C. (2013). Activation of Nrf2-ARE signal pathway in hippocampus of amygdala kindling rats. Neurosci. Lett..

[36] Cheng Y., Luo F., Zhang Q., Sang Y., Chen X., Zhang L., Liu Y., Li X., Li J., Ding H. (2018). α-Lipoic acid alleviates pentetrazol-induced neurological deficits and behavioral dysfunction in rats with seizures via an Nrf2 pathway. RSC Adv..

[37] Zhang Y.N., Dong H.T., Yang F.B., Wang Z.Q., Ma Z.H., Ma S.Z., Ma X.D., Duan L. (2018). Nrf2-ARE signaling pathway regulates the expressions of A1R and ENT1 in the brain of epileptic rats. Eur. Rev. Med. Pharmacol. Sci..

[38] Liu Z., Yang C., Meng X., Li Z., Lv C., Cao P. (2018). Neuroprotection of edaravone on the hippocampus of kainate-induced epilepsy rats through Nrf2/HO-1 pathway. Neurochem. Int..

[39] Oswald M.C.W., Garnham N., Sweeney S.T., Landgraf M. (2018). Regulation of neuronal development and function by ROS. FEBS Letters.

[40] Son Y., Cheong Y.K., Kim N.H., Chung H.T., Kang D.G., Pae H.O. (2011). Mitogen-activated protein kinases and reactive oxygen species: How can ROS activate MAPK pathways?. J. Signal Transduct..

[41] Gloire G., Legrand-Poels S., Piette J. (2006). NF-ΚB activation by reactive oxygen species: Fifteen years later. Biochem. Pharmacol..

[42] Schreck R., Rieber P., Baeuerle P.A. (1991). Reactive oxygen intermediates as apparently widely used messengers in the activation of the NF-Kappa B transcription factor and HIV-1. EMBO J..

[43] Vezzani A., Balosso S., Ravizza T. (2019). Neuroinflammatory pathways as treatment targets and biomarkers in epilepsy. Nat. Rev. Neurol..

[44] Webster K.M., Sun M., Crack P., O’Brien T.J., Shultz S.R., Semple B.D. (2017). Inflammation in epileptogenesis after traumatic brain injury. J. Neuroinflamm..

[45] Eastman C.L., D’Ambrosio R., Ganesh T. (2020). Modulating neuroinflammation and oxidative stress to prevent epilepsy and improve outcomes after traumatic brain injury. Neuropharmacology.

[46] Bajwa E., Pointer C.B., Klegeris A. (2019). The role of mitochondrial damage-associated molecular patterns in chronic neuroinflammation. Mediat. Inflamm..

[47] Lugrin JRosenblatt-Velin N., Parapanov R., Liaudet L. (2014). The role of oxidative stress during inflammatory processes. Biol. Chem..

[48] Maroso M., Balosso S., Ravizza T., Liu J., Aronica E., Iyer A., Rossetti C., Molteni M., Casalgrandi M., Manfredi A.A. (2010). Toll-like receptor 4 and high-mobility group box-1 are involved in ictogenesis and can be targeted to reduce seizures. Nat. Med..

[49] Vezzani B., Carinci M., Patergnani S., Pasquin M.P., Guarino A., Aziz N., Pinton P., Simonato M., Giorgi C. (2020). The dichotomous role of inflammation in the CNS: A mitochondrial point of view. Biomolecules..

[50] Shi Y., Zhang L., Teng J., Miao W. (2018). HMGB1 mediates microglia activation via the TLR4/NF-ΚB pathway in coriaria lactone induced epilepsy. Mol. Med. Rep..

[51] Dai S., Zheng Y., Wang Y., Chen Z. (2021). HMGB1, neuronal excitability and epilepsy. Acta Epileptol..

[52] Victor T.R., Tsirka S.E. (2020). Microglial contributions to aberrant neurogenesis and pathophysiology of epilepsy. Neuroimmunol. Neuroinflamm..

[53] McElroy P.B., Liang L.P., Day B.J., Patel M. (2017). Scavenging reactive oxygen species inhibits status epilepticus-induced neuroinflammation. Exp. Neurol..

[54] Badawi G.A., Shokr M.M., Zaki H.F., Mohamed A.F. (2021). Pentoxifylline prevents epileptic seizure via modulating HMGB1/RAGE/TLR4 signalling pathway and improves memory in pentylenetetrazol kindling rats. Clin. Exp. Pharmacol. Physiol..

[55] De Deus J.L., Amorim M.R., de Barcellos Filho P.C.G., de Oliveira J.A.C., Batalhão M.E., Garcia-Cairasco N., Cárnio E.C., Leão R.M., Branco L.G.S., Cunha A.O.S. (2020). Inflammatory markers in the hippocampus after audiogenic kindling. Neurosci. Lett..

[56] Terrone G., Balosso S., Pauletti A., Ravizza T., Vezzani A. (2020). Inflammation and reactive oxygen species as disease modifiers in epilepsy. Neuropharmacology.

[57] Terrone G., Frigerio F., Balosso S., Ravizza T., Vezzani A. (2019). Inflammation and reactive oxygen species in status epilepticus: Biomarkers and implications for therapy. Epilepsy Behav..

[58] Xia L., Pan S.Q., Zhang Q.M., Zhou Q., Xia L., Lu Z.N. (2018). Elevated IL-6 and IL-1β are associated with temporal lobe epilepsy: A study in chinese patients. Eur. J. Inflamm..

[59] Ethemoglu O., Ay H., Koyuncu I., Gonel A. (2018). Comparison of cytokines and prooxidants/antioxidants markers among adults with refractory versus well-controlled epilepsy: A cross-sectional study. Seizure.

[60] Pecoraro-Bisogni F., Lignani G., Contestabile A., Castroflorio E., Pozzi D., Rocchi A., Prestigio C., Orlando M., Valente P., Massacesi M. (2018). REST-Dependent presynaptic homeostasis induced by chronic neuronal hyperactivity. Mol. Neurobiol..

[61] Turrigiano G.G., Nelson S.B. (2004). Homeostatic plasticity in the developing nervous system. Nat. Rev. Neurosci..

[62] Guarnieri F.C., Pozzi D., Raimondi A., Fesce R., Valente M.M., Delvecchio V.S., Van Esch H., Matteoli M., Benfenati F., D’Adamo P. (2017). A novel SYN1 missense mutation in non-syndromic X-linked intellectual disability affects synaptic vesicle life cycle, clustering and mobility. Hum. Mol. Genet..

[63] Valente P., Castroflorio E., Rossi P., Fadda M., Sterlini B., Cervigni R.I., Prestigio C., Giovedì S., Onofri F., Mura E. (2016). PRRT2 is a key component of the Ca(2+)-dependent neurotransmitter release machinery. Cell Rep..

[64] Buckmaster P.S., Yamawaki R., Thind K. (2016). More docked vesicles and larger active zones at basket cell-to-granule cell synapses in a rat model of temporal lobe epilepsy. J. Neurosci..

[65] Colasante G., Qiu Y., Massimino L., Di Berardino C., Cornford J.H., Snowball A., Weston M., Jones S.P., Giannelli S., Lieb A. (2020). In vivo CRISPRa decreases seizures and rescues cognitive deficits in a rodent model of epilepsy. Brain.

[66] Salpietro V., Dixon C.L., Guo H., Bello O.D., Vandrovcova J., Efthymiou S., Maroofian R., Heimer G., Burglen L., Valence S. (2019). AMPA receptor GluA2 subunit defects are a cause of neurodevelopmental disorders. Nat. Commun..

[67] Smith H.L., Bourne J.N., Cao G., Chirillo M.A., Ostroff L.E., Watson D.J., Harris K.M. (2016). Mitochondrial support of persistent presynaptic vesicle mobilization with age-dependent synaptic growth after LTP. Elife.

[68] Augustin K., Khabbush A., Williams S., Eaton S., Orford M., Cross J.H., Heales S.J.R., Walker M.C., Williams R.S.B. (2018). Mechanisms of action for the medium-chain triglyceride ketogenic diet in neurological and metabolic disorders. Lancet Neurol..

[69] Maru E., Kanda M., Ashida H. (2002). Functional and morphological changes in the hippocampal neuronal circuits associated with epileptic seizures. Epilepsia.

[70] Repetto D., Camera P., Melani R., Morello N., Russo I., Calcagno E., Tomasoni R., Bianchi F., Berto G., Giustetto M. (2014). P140Cap regulates memory and synaptic plasticity through Src-mediated and citron-n-mediated actin reorganization. J. Neurosci..

[71] Südhof T.C. (2012). The presynaptic active zone. Neuron.

[72] Sweatt J.D. (2016). Dynamic DNA methylation controls glutamate receptor trafficking and synaptic scaling. J. Neurochem..

[73] Clayton D.F., Anreiter I., Aristizabal M., Frankland P.W., Binder E.B., Citri A. (2020). The role of the genome in experience-dependent plasticity: Extending the analogy of the genomic action potential. Proc. Natl. Acad. Sci. USA.

[74] Tien N.W., Kerschensteiner D. (2018). Homeostatic plasticity in neural development. Neural Dev..

[75] Méndez-Armenta M., Nava-Ruíz C., Juárez-Rebollar D., Rodríguez-Martínez E., Gómez P.Y. (2014). Oxidative stress associated with neuronal apoptosis in experimental models of epilepsy. Oxid. Med. Cell. Longev..

[76] Hoffmann S., Orlando M., Andrzejak E., Bruns C., Trimbuch T., Rosenmund C., Garner C.C., Ackermann F. (2019). Light-activated ROS production induces synaptic autophagy. J. Neurosci..

[77] Sears S.M., Hewett S.J. (2021). Influence of glutamate and GABA transport on brain excitatory/inhibitory balance. Exp. Biol. Med..

[78] Peterson A.R., Binder D.K. (2019). Regulation of synaptosomal GLT-1 and GLAST during epileptogenesis. Neuroscience.

[79] Schijns O.E., Bisschop J., Rijkers K., Dings J., Vanherle S., Lindsey P., Smeets H.J., Hoogland G. (2020). GAT-1 (rs2697153) and GAT-3 (rs2272400) polymorphisms are associated with febrile seizures and temporal lobe epilepsy. Epileptic Disord..

[80] Petroff O.A. (2002). GABA and glutamate in the human brain. Neuroscientist.

[81] During M.J., Spencer D.D. (1993). Extracellular hippocampal glutamate and spontaneous seizure in the conscious human brain. Lancet.

[82] Eid T., Williamson A., Lee T.S., Petroff O.A., de Lanerolle N.C. (2008). Glutamate and astrocytes—key players in human mesial temporal lobe epilepsy?. Epilepsia.

[83] Rae C., Moussa C.e.l.-H., Griffin J.L., Parekh S.B., Bubb W.A., Hunt N.H., Balcar V.J. (2006). A metabolomic approach to ionotropic glutamate receptor subtype function: A nuclear magnetic resonance in vitro investigation. J. Cereb. Blood Flow Metab..

[84] Eid T., Gruenbaum S.E., Dhaher R., Lee T.W., Zhou Y., Danbolt N.C. (2016). The glutamate-glutamine cycle in epilepsy. Adv. Neurobiol..

[85] Hanada T. (2020). Ionotropic glutamate receptors in epilepsy: A review focusing on AMPA and NMDA receptors. Biomolecules.

[86] Danbolt N.C. (2001). Glutamate uptake. Prog. Neurobiol..

[87] Vishnoi S., Raisuddin S., Parvez S. (2016). Glutamate excitotoxicity and oxidative stress in epilepsy: Modulatory role of melatonin. J. Environ Pathol Toxicol. Oncol.

[88] Peng W.F., Ding J., Li X., Fan F., Zhang Q.Q., Wang X. (2016). N-methyl-d-aspartate receptor NR2B subunit involved in depression-like behaviours in lithium chloride-pilocarpine chronic rat epilepsy model. Epilepsy Res..

[89] Chen K., Baram T.Z., Soltesz I. (1999). Febrile seizures in the developing brain result in persistent modification of neuronal excitability in limbic circuits. Nat. Med..

[90] Barker-Haliski M., White H.S. (2015). Glutamatergic mechanisms associated with seizures and epilepsy. Cold Spring Harb Perspect. Med..

[91] Levite M., Zelig D., Friedman A., Ilouz N., Eilam R., Bromberg Z., Lasu A.A.R., Arbel-Alon S., Edvardson S., Tarshish M. (2020). Dual-targeted autoimmune sword in fatal epilepsy: Patient’s glutamate receptor AMPA GluR3B peptide autoimmune antibodies bind, induce reactive oxygen species (ROS) in, and kill both human neural cells and T cells. J. Autoimmun..

[92] Schousboe A., Scafidi S., Bak L.K., Waagepetersen H.S., McKenna M.C. (2014). Glutamate metabolism in the brain focusing on astrocytes. Adv. Neurobiol..

[93] Patel D.C., Tewari B.P., Chaunsali L., Sontheimer H. (2019). Neuron-glia interactions in the pathophysiology of epilepsy. Nat. Rev. Neurosci..

[94] Twible C., Abdo R., Zhang Q. (2021). Astrocyte role in temporal lobe epilepsy and development of mossy fiber sprouting. Front. Cell. Neurosci..

[95] Proper E.A., Hoogland G., Kappen S.M., Jansen G.H., Rensen M.G., Schrama L.H., van Veelen C.W., van Rijen P.C., van Nieuwenhuizen O., Gispen W.H. (2002). Distribution of glutamate transporters in the hippocampus of patients with pharmaco-resistant temporal lobe epilepsy. Brain.

[96] Nikolic L., Nobili P., Shen W., Audinat E. (2020). Role of astrocyte purinergic signaling in epilepsy. Glia.

[97] Nikolic L., Shen W., Nobili P., Virenque A., Ulmann L., Audinat E. (2018). Blocking TNFα-driven astrocyte purinergic signaling restores normal synaptic activity during epileptogenesis. Glia.

[98] Greenfield Jr L.J. (2013). Molecular mechanisms of antiseizure drug activity at GABAA receptors. Seizure.

[99] Ghit A., Assal D., Al-Shami A.S., Hussein D.E.E. (2021). GABAA receptors: Structure, function, pharmacology, and related disorders. J. Genet. Eng. Biotechnol..

[100] McDonald J.W., Garofalo E.A., Hood T., Sackellares J.C., Gilman S., McKeever P.E., Troncoso J.C., Johnston M.V. (1991). Altered excitatory and inhibitory amino acid receptor binding in hippocampus of patients with temporal lobe epilepsy. Ann. Neurol..

[101] Johnson E.W., de Lanerolle N.C., Kim J.H., Sundaresan S., Spencer D.D., Mattson R.H., Zoghbi S.S., Baldwin R.M., Hoffer P.B., Seibyl J.P. (1992). Central and peripheral benzodiazepine receptors: Opposite changes in human epileptogenic tissue. Neurology.

[102] Savic I., Persson A., Roland P., Pauli S., Sedvall G., Widén L. (1988). In-vivo demonstration of reduced benzodiazepine receptor binding in human epileptic foci. Lancet.

[103] Henry T.R., Frey K.A., Sackellares J.C., Gilman S., Koeppe R.A., Brunberg J.A., Ross D.A., Berent S., Young A.B., Kuhl D.E. (1993). In vivo cerebral metabolism and central benzodiazepine-receptor binding in temporal lobe epilepsy. Neurology.

[104] Amato A., Connolly C.N., Moss S.J., Smart T.G. (1999). Modulation of neuronal and recombinant GABAA receptors by redox reagents. J. Physiol..

[105] Pan Z.H., Zhang X., Lipton S.A. (2000). Redox modulation of recombinant human GABAA receptors. Neuroscience.

[106] Accardi M.V., Daniels B.A., Brown P.M.G.E., Fritschy J.M., Tyagarajan S.K., Bowie D. (2014). Mitochondrial reactive oxygen species regulate the strength of inhibitory GABA-mediated synaptic transmission. Nat. Commun..

[107] Frantseva M.V., Perez J.L., Carlen P.L. (1998). Changes in membrane and synaptic properties of thalamocortical circuitry caused by hydrogen peroxide. J. Neurophysiol..

[108] Akyuz E., Polat A.K., Eroglu E., Kullu I., Angelopoulou E., Paudel Y.N. (2021). Revisiting the role of neurotransmitters in epilepsy: An updated review. Life Sci..

[109] Kalilani L., Sun X., Pelgrims B., Noack-Rink M., Villanueva V. (2018). The epidemiology of drug-resistant epilepsy: A systematic review and meta-analysis. Epilepsia.

[110] Steriade C., French J., Devinsky O. (2020). Epilepsy: Key experimental therapeutics in early clinical development. Exp. Opin. Investig. Drugs.

[111] Nazıroğlu M., Yürekli V.A. (2013). Effects of antiepileptic drugs on antioxidant and oxidant molecular pathways: Focus on trace elements. Cell. Mol. Neurobiol..

[112] Mahle C., Dasgupta A. (1997). Decreased total antioxidant capacity and elevated lipid hydroperoxide concentrations in sera of epileptic patients receiving phenytoin. Life Sci..

[113] Liu C.S., Wu H.M., Kao S.H., Wei Y.H. (1997). Phenytoin-mediated oxidative stress in serum of female epileptics: A possible pathogenesis in the fetal hydantoin syndrome. Hum. Exp. Toxicol..

[114] Ficarra S., Misiti F., Russo A., Carelli-Alinovi C., Bellocco E., Barreca D., Laganà G., Leuzzi U., Toscano G., Giardina B. (2013). Antiepileptic carbamazepine drug treatment indices alteration of membrane in red blood cells: Possible positive effects on metabolism and oxidative stress. Biochimie.

[115] Gathwala G., Marwah A., Gahlaut V., Marwah P. (2011). Effect of high-dose phenobarbital on oxidative stress in perinatal asphyxia: An open label randomized controlled trial. Indian Pediatr..

[116] Rajasekaran K. (2005). Seizure-induced oxidative stress in rat brain regions: Blockade by nNOS inhibition. Pharmacol. Biochem. Behav..

[117] Krauss G.L., Klein P., Brandt C., Lee S.K., Milanov I., Milovanovic M., Steinhoff B.J., Kamin M. (2020). Safety and efficacy of adjunctive cenobamate (YKP3089) in patients with uncontrolled focal seizures: A multicentre, double-blind, randomised, placebo-controlled, dose-response trial. Lancet Neurol..

[118] Wiciński M., Puk O., Malinowski B. (2021). Cenobamate: Neuroprotective potential of a new antiepileptic drug. Neurochem. Res..

[119] Kumar P., Kalonia H., Kumar A. (2012). Possible GABAergic mechanism in the neuroprotective effect of gabapentin and lamotrigine against 3-nitropropionic acid induced neurotoxicity. Eur. J. Pharmacol..

[120] Devinsky O., Marsh E., Friedman D., Thiele E., Laux L., Sullivan J., Miller I., Flamini R., Wilfong A., Filloux F. (2016). Cannabidiol in patients with treatment-resistant epilepsy: An open-label interventional trial. Lancet Neurol..

[121] Gray R.A., Whalley B.J. (2020). The proposed mechanisms of action of CBD in epilepsy. Epileptic Disord..

[122] Atalay S., Jarocka-Karpowicz I., Skrzydlewska E. (2019). Antioxidative and anti-inflammatory properties of cannabidiol. Antioxidants.

[123] Shakeel S., Rehman M.U., Tabassum N., Amin U., Mir M.U.R. (2017). Effect of Naringenin (a naturally occurring flavanone) against pilocarpine-induced status epilepticus and oxidative stress in mice. Pharmacogn. Mag..

[124] Tawfik M.K. (2011). Coenzyme Q10 enhances the anticonvulsant effect of phenytoin in pilocarpine-induced seizures in rats and ameliorates phenytoin-induced cognitive impairment and oxidative stress. Epilepsy Behav..

[125] Shin E.J., Suh S.K., Lim Y.K., Hjelle O.P., Ottersen O.P., Shin C.Y., Ko K.H., Kim W.-K., Kim D.S., Chun W. (2005). Ascorbate attenuates trimethyltin-induced oxidative burden and neuronal degeneration in the rat hippocampus by maintaining glutathione homeostasis. Neuroscience.

[126] Dhir A. (2018). Curcumin in epilepsy disorders: Curcumin and epilepsy. Phytother. Res..

[127] Mehvari J., Motlagh F.G., Najafi M., Ghazvini M.R.A., Naeini A.A., Zare M. (2016). Effects of vitamin E on seizure frequency, electroencephalogram findings, and oxidative stress status of refractory epileptic patients. Adv. Biomed. Res..

[128] Wang W., Wu Y., Zhang G., Fang H., Wang H., Zang H., Xie T., Wang W. (2014). Activation of Nrf2-ARE signal pathway protects the brain from damage induced by epileptic seizure. Brain Res..

[129] Kenney-Jung D., Vezzani A., Kahoud R.J., LaFrance-Corey R.G., Ho Mai-Lan Muskardin T.W., Wirrell E.C., Howe C.L., Payne E.T. (2016). Febrile infection- related epilepsy syndrome treated with anakinra. Ann. Neurol..

[130] Dilena R., Mauri E., Aronica E., Bernasconi P., Bana C., Cappelletti C., Carrabba G., Ferrero S., Giorda R., Guez S. (2019). Therapeutic effect of anakinra in the relapsing chronic phase of febrile infection–related epilepsy syndrome. Epilepsia Open.

[131] Jyonouchi H., Geng L. (2016). Intractable epilepsy (IE) and responses to anakinra, a human recombinant IL-1 receptor antagonist (IL-1Ra): Case reports. J. Clin. Cell. Immunol..

[132] DeSena A.D., Do T., Schulert G.S. (2018). Systemic autoinflammation with intractable epilepsy managed with interleukin-1 blockade. J. Neuroinflamm..

[133] Jun J.S., Lee S.T., Kim R., Chu K., Lee S.K. (2018). Tocilizumab treatment for new onset refractory status epilepticus. Ann. Neurol..

[134] Cantarín-Extremera V., Jiménez-Legido M., Duat-Rodríguez A., García-Fernández M., Ortiz-Cabrera N.V., Ruiz-Falcó-Rojas M.L., González-Gutiérrez-Solana L. (2020). Tocilizumab in pediatric refractory status epilepticus and acute epilepsy: Experience in two patients. J. Neuroimmunol..

[135] Nowak M., Strzelczyk A., Reif P.S., Schorlemmer K., Bauer S., Norwood B.A., Oertel W.H., Rosenow F., Strik H., Hamer H.M. (2012). Minocycline as potent anticonvulsivant in a patient with astrocytoma and drug resistant epilepsy. Seizure.

[136] Lagarde S., Villeneuve N., Trébuchon A., Kaphan E., Lepine A., McGonigal A., Roubertie A., Barthez M.J., Trommsdorff V., Lefranc J. (2016). Anti-tumor necrosis factor alpha therapy (adalimumab) in Rasmussen’s encephalitis: An open pilot study. Epilepsia.

[137] Lance E.I., Sreenivasan A.K., Zabel T.A., Kossoff E.H., Comi A.M. (2013). Aspirin use in Sturge- Weber syndrome: Side effects and clinical outcomes. J. Child Neurol..

[138] Godfred R.M., Parikh M.S., Haltiner A.M., Caylor L.M., Sepkuty J.P., Doherty M.J. (2013). Does aspirin use make it harder to collect seizures during elective video- EEG telemetry?. Epilepsy Behav..

[139] Bialer M., Johannessen S.I., Levy R.H., Perucca E., Tomson T., HWhite S. (2013). Progress report on new antiepileptic drugs: A summary of the Eleventh Eilat Conference (EILAT XI). Epilepsy Res..

[140] Neal E.G., Chaffe H., Schwartz R.H., Lawson M.S., Edwards N., Fitzsimmons G., Whitney A., Cross J.H. (2008). The ketogenic diet for the treatment of childhood epilepsy: A randomised controlled trial. Lancet Neurol..

[141] Simeone T.A., Matthews S.A., Samson K.K., Simeone K.A. (2017). Regulation of brain PPARgamma2 contributes to ketogenic diet anti-seizure efficacy. Exp. Neurol..

[142] Knowles S., Budney S., Deodhar M., Matthews S.A., Simeone K.A., Simeone T.A. (2018). Ketogenic diet regulates the antioxidant catalase via the transcription factor PPARγ2. Epilepsy Res..

[143] Shimazu T., Hirschey M.D., Newman J., He W., Shirakawa K., Le Moan N., Grueter C.A., Lim H., Saunders L.R., Stevens R.D. (2013). Suppression of oxidative stress by -hydroxybutyrate, an endogenous histone deacetylase inhibitor. Science.

[144] Dalle-Donne I., Scaloni A., Giustarini D., Cavarra E., Tell G., Lungarella G., Colombo R., Rossi R., Milzani A. (2005). Proteins as biomarkers of oxidative/nitrosative stress in diseases: The contribution of redox proteomics. Mass Spectrom. Rev..

[145] Farah M.E., Sirotkin V., Haarer B., Kakhniashvili D., Amberg D.C. (2011). Diverse protective roles of the actin cytoskeleton during oxidative stress. Cytoskeleton.

[146] Hidalgo C., Carrasco M.A., Muñoz P., Núñez M.T. (2007). A role for reactive oxygen/nitrogen species and iron on neuronal synaptic plasticity. Antioxid. Redox Signal..

[147] Ulasov A.V., Rosenkranz A.A., Georgiev G.P., Sobolev A.S. (2021). Nrf2/Keap1/ARE signaling: Towards specific regulation. Life Sci..

[148] Clarke J.D., Hsu A., Williams D.E., Dashwood R.H., Stevens J.F., Yamamoto M., Ho E. (2011). Metabolism and tissue distribution of sulforaphane in Nrf2 knockout and wild-type mice. Pharm. Res..

[149] Shekh-Ahmad T., Eckel R., Naidu S.D., Higgins M., Yamamoto M., Dinkova-Kostova A.T., Kovac S., Abramov A.Y., Walker M.C. (2018). KEAP1 inhibition is neuroprotective and suppresses the development of epilepsy. Brain.

[150] Lynch D.R., Chin M.P., Delatycki M.B., Subramony S.H., Corti M., Hoyle J.C., Boesch S., Nachbauer W., Mariotti C., Mathews K.D. (2021). Safety and efficacy of Omaveloxolone in Friedreich ataxia (MOXIe Study). Ann. Neurol..

[151] Shekh-Ahmad T., Lieb A., Kovac S., Gola L., Wigley C., Abramov A.Y., Walker M.C. (2019). Combination antioxidant therapy prevents epileptogenesis and modifies chronic epilepsy. Redox. Biol..

[152] Lin T.K., Chen S.D., Lin K.J., Yao-Chung C. (2020). Seizure-Induced Oxidative Stress in Status Epilepticus: Is Antioxidant Beneficial?. Antioxidants.

[153] Chang S.J., Yu B.C. (2010). Mitochondrial matters of the brain: Mitochondrial dysfunction and oxidative status in epilepsy. J. Bioenerg. Biomembr..

[154] Witt J.A., Helmstaedter C. (2017). Cognition in epilepsy: Current clinical issues of interest. Curr. Opin. Neurol..

